# Getting Rigor Right: A Framework for Methodological Choice in Adaptive Monitoring and Evaluation

**DOI:** 10.9745/GHSP-D-22-00243

**Published:** 2023-12-18

**Authors:** Christina Synowiec, Erin Fletcher, Luke Heinkel, Taylor Salisbury

**Affiliations:** a Results for Development, Washington, DC, USA.

## Abstract

The field of global development has embraced the idea that programs require agile, adaptive approaches to monitoring, evaluation, and learning. But considerable debate still exists around which methods are most appropriate for adaptive learning. Researchers have a range of proven and novel tools to promote a culture of adaptation and learning. These tools include lean testing, rapid prototyping, formative research, and structured experimentation, all of which can be utilized to generate responsive feedback (RF) to improve social change programs. With such an extensive toolkit, how should one decide which methods to employ? In our experience, the level of rigor used should be responsive to the team's level of certainty about the program design being investigated—how certain—or confident—are we that a program design will produce its intended results? With less certainty, less rigor is needed; with more certainty, more rigor is needed. In this article, we present a framework for getting rigor right and illustrate its use in 3 case studies. For each example, we describe the feedback methods used and why, how the approach was implemented (including how we conducted cocreation and ensured buy-in), and the results of each engagement. We conclude with lessons learned from these examples and how to use the right kind of RF mechanism to improve social change programs.

## Abstract (precis)

The authors present a framework using the level of certainty in a program's design to choose the appropriate level of rigor for adaptive learning activities conducted during program implementation.

## Teaser

**Key Findings:**

- The level of certainty about an entire program or smaller elements of a program should correlate with the level of rigor used for adaptive learning.
- Cocreation is critical to ensuring right-fit rigor and that results and data generated are useful to decision-makers.
- Certainty is dynamic over the course of an engagement, and the assessment of it should be revisited on an ongoing basis following learning activities.

**Key Implications:**

- Our framework can guide stakeholders through the process of assessing their programs to design relevant, timely, and iterative adaptive learning or responsive feedback activities.
- Future case studies should inform continuous adaptation and iteration of the framework to reflect the experiences of research and development practitioners.

## INTRODUCTION

The field of global development has embraced the idea that programs require agile, adaptive approaches to monitoring, evaluation, and learning (MEL). This is an evolution in how the development community has previously approached MEL. There is increased recognition that development programs are complex and operate within broader systems that are ever-changing. Adaptive learning and measurement approaches respond to the inherent mismatch between linear, static approaches to program design and implementation. In addition to holding programs accountable for results, there is interest in using data and evidence for ongoing learning. However, despite advances in methodology and a growing consensus around the ultimate value of a learning-focused approach, there is still considerable debate around which methods are most appropriate for adaptive learning. There can be a seeming tension between doing research rigorously and doing research rapidly, or, for example, relevance to programmatic learning versus donor accountability.

The solution to this tension is a focus on right-fit monitoring and evaluation (M&E) systems, utilization-focused evaluations, and responsive feedback (RF) mechanisms—generating evidence that will be used for decision-making and ultimately improve opportunities for program impact—or adaptive learning.^1^^,^^2^ As with all fields in development, there has been a proliferation in terminology around this concept, reflecting a growing movement to intentionally use strategies and actions to critically reflect on and analyze data, information, and knowledge on an ongoing basis to inform decisions. Other terms that align around these principles include MEL,^3^ RF mechanisms,^2^ and adaptive learning.^4^ We do not take a stance on the value of one approach over the other but use the term adaptive learning throughout for consistency and clarity. The goal is to address the use of “wrong-fit” systems, which use valuable program resources and may not provide useful data for decision-making.

Research practitioners have a range of approaches within their reach to promote a culture of adaptation and learning. These methods take various names, from lean testing^5^ to rapid prototyping,^6^ from formative research to structured experimentation^7^—regardless of nomenclature, the goal of each is to generate RF to improve social change programs. But with such a range of methods, what is the methodology for selecting the best approach?

We propose that the appropriate level of rigor employed should be determined by the level of certainty about a program's design: the less certain, the less rigor needed, and vice versa. In this article, we present a framework that relies on 4 dimensions of certainty to determine the appropriate level of rigor (building on another rigor framework^8^). We apply the framework to 3 case studies and conclude with lessons learned and suggestions for implementation. We define rigor—in the context of development interventions—as our level of confidence in a research method's ability to determine causality. With higher-rigor methods, we have greater confidence that observed changes in behavior are caused by an intervention. With lower-rigor methods, we have less confidence that changes in behavior are caused by an intervention. By defining rigor in this way, we consciously avoid taking a hard stance on the traditional debate about the validity of quantitative versus qualitative methods. In our view, both sets of methods are relevant, both are important, and both can be equally rigorous or not rigorous depending on how they are deployed. What matters is a method's ability to tell us something about causality.

We present a framework that relies on 4 dimensions of certainty to determine the appropriate level of rigor for a program's design.

Still, we do view methods as falling into a rough hierarchy of rigor, with randomized controlled trials (RCTs) and regression discontinuity designs slotting in higher than case studies and lean testing. This hierarchy is not rigid—most methods overlap in rigor depending on how researchers put them into practice. But, all else being equal, impact evaluation methods like RCTs and regression discontinuity designs that rely on (quasi-) randomization and large sample sizes will typically give us more confidence in the results' linkage to causality than methods that do not include these design elements.

## EXISTING LITERATURE ON FRAMEWORKS FOR CHOOSING ADAPTIVE LEARNING METHODS

We summarize the growing body of literature that codifies frameworks for choosing appropriate adaptive learning or RF methods. We are by no means the first to tout the advantages of these methods; we build off this existing pivot in development research to formally link the level of certainty in program design with the level of rigor in methods used.^9^

Andrews et al. are among the first to have outlined the principles of problem-driven iterative adaptation in the scholarly literature and convey its benefits in fostering learning and improving programming.^10^ Along with others such as The Curve,^11^ Andrews et al. describe how incorporating feedback loops into program designs—designs built on real-world experimentation and active learning—can generate dynamic and responsive programming. Pritchett et al. bring multiple adaptive learning ideas together as part of a history that describes the evolution of M&E approaches, suggesting that an era of experimentation—as opposed to RCTs—should define development research moving forward.^7^ Gugerty and Karlan offer a set of principles for engaging in evaluation that does not meet rigorous standards of causal inference, highlighting areas where a causal impact estimate is not appropriate.^12^ However, they do not elaborate on methods or alternatives.

More recent literature attempts to provide frameworks for making decisions about how to implement RF methods. Braverman et al. outline the components of methodological rigor and detail factors that drive decisions about methodological rigor, such as budget, time, and measurement strategies.^14^ Others seek to evaluate the value of mixed methods, highlighting the role of qualitative data collection and qualitative evaluation methods.^13^ Aston and Apgar and Eckhardt and DeVon provide frameworks to support evaluators to effectively combine M&E methods to take a right-fit approach.^15^^,^^16^ Ramalingam and Buffardi outline methods for collecting data for adaptive or experimental methods in a way that ensures high data quality, appropriate investment in monitoring, and strengthened capacities.^17^ In the “CART” principles for engagement, Gugerty and Karlan suggest that data collected should be credible, actionable, responsible, and transportable.^18^ The framework emphasizes an ethical lens for research and adaptive methods. But it may provide too high of a barrier to entry for adaptive learning given the focus on unbiasedness and the presence of a counterfactual. Our aim in this article is to build on the existing literature by providing a useful tool to identify the right level of rigor to answer a given research question and examples of how to apply the learning that emerges toward evidence-based decision-making.

## PROPOSED FRAMEWORK FOR GETTING RIGOR RIGHT

**FIGURE 1:**
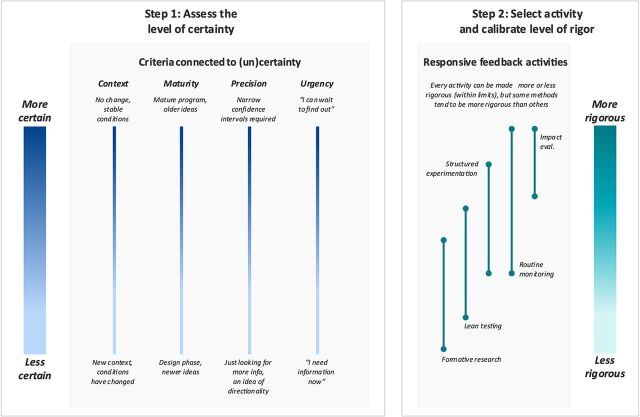
A Framework for Connecting Certainty to Rigor Abbreviation: eval., evaluation.

We visualize in Figure 1 our framework for choosing the level of rigor—from less rigorous to more rigorous—in adaptive learning activities based on the experience of our team at Results for Development (R4D). In the following section, we present the framework in practice via 3 case studies.

### Step 1: Assess the Level of Certainty

The first step in choosing rigor is to assess how certain—or confident—we are that a program design will produce its intended results. Certainty in program design is connected to and influenced by several factors, including the quality and comprehensiveness of existing evidence, the characteristics of the program, and the program's context. It is also influenced by the type of intended results we are interested in. In some cases, we may be interested in the shorter-term, intermediate outcomes identified in a theory of change (TOC). While in others, we may be more interested in longer-term outcomes or impact. At the outset of a learning engagement, we work with partners to collectively identify and prioritize the learning questions that will guide learning about the intended results—something that generally happens through generating or revising the program TOC. We then begin the process of assessing certainty and rigor based on the prioritized questions. Above all, our goal is to match the adaptive learning method to the learning question. Rather than start with the method in mind, we work from the learning question and the certainty we have about the way a program design works and only then consider what method is appropriate.

The first step in choosing rigor is to assess how certain—or confident—we are that a program design will produce its intended results.

We propose 4 criteria that have consistently informed our methodological choices since we began piloting adaptive learning methods in 2015: context, maturity, precision, and urgency (Table 1). Each is associated with certainty along a continuum (Figure 1). To operationalize them, we need to decide where a program is along the criteria continuum, a process akin to “setting the dials” on a control panel or on a radio. This step should involve consultations with a wide range of stakeholders to determine what data would be most useful to the decision-makers, as well as the key learning questions. Once the dials are set, our team then triangulates between all the criteria to settle on our overall assessment of certainty and identify a “best-fit line” that runs through our criteria.

**TABLE 1.** Criteria for Assessing a Program's Certainty in Achieving Intended Outcomes

Criterion and Rationale	Example Questions	Interpretation
**Context**: The context where an implementer implements an intervention has many and varied effects on intervention outcomes. When an implementer knows the context well, they can anticipate how it will affect their implementation activities. This predictability in how context influences an intervention influences certainty that the design of an intervention is appropriate for the context.	• How similar or different is the setting for a program compared to the setting of previous program activities? • How much has the context changed over time? • Are conditions stable, or have they changed recently?	**Increased certainty**: More similar settings and more stable conditions (less change in the context) mean more predictable outcomes, which increases certainty. **Decreased certainty**: Less similar setting and less stable conditions (more change in the context) mean less predictable outcomes, which decreases certainty.
**Maturity**: An implementer can more accurately anticipate how program design changes will influence outcomes when they have more experience carrying out the program or when the elements of the program design are older and more familiar to them.	• How much experience does the implementer have in implementing the existing program design?	**Increased certainty**: With older, more mature programs, implementers have greater experience and familiarity with how they work in practice, which increases certainty. **Decreased certainty**: With newer, less mature programs, implementers have less experience and familiarity with how they work in practice, which decreases certainty.
**Precision**: Decision-makers have different standards for the robustness of the evidence they take into consideration when making decisions. Sometimes they want to be very certain in the evidence they are considering, while other times they are okay if the evidence requires a lot of caveats. From a research perspective, this translates into confidence intervals. If those intervals can be wider, the goal of the research would then be less about obtaining exact point estimates and more about understanding directionality (i.e., can we generally expect a positive outcome or a negative outcome?). The precision criterion is typically more salient when assessing the use of quantitative research methods.	• How precise do the estimates of change caused by the adaptive learning activity need to be? • Do the confidence intervals on the estimate need to be very narrow—and therefore very defensible from a statistical point of view? • Or can they be wider—and less defensible?	**Increased certainty**: The narrower confidence intervals that come with more precision increases certainty. **Decreased certainty:** The wider confidence intervals that come with less precision decreases certainty.
**Urgency**: Evidence generated through a learning activity will ideally be used to inform key decisions about program implementation. How soon those decisions must be made will dictate how certain we can be that a program's design has fully run its course and produced its intended results.	• How soon does the adaptive learning activity need to produce results? • Can the implementer wait for a year to receive the results? • Or will the implementer be making a decision in 1 month and so needs information as soon as possible?	**Increased certainty**: Less urgency increases certainty because there will be more time to measure the outcomes from design changes. **Decreased certainty**: More urgency decreases certainty because there will be less time to measure the outcomes from design changes.

We do not propose any weighting scheme for these criteria. The relative importance of each criterion compared to the others will depend on the research and learning questions identified. In some cases, a single criterion may trump the others, leaving researchers with less flexibility to consider the other criteria. For example, if a partner needs results in 3 months, the methods that can be deployed on that short of a time frame are always going to be less rigorous regardless of whether the “dials” are set high on the other criteria (i.e., even if the partner wants very precise results, you simply cannot implement an RCT and produce results in 3 months).^19^ In addition, in our experience applying the criteria should involve a mix of stakeholders—ranging from researchers to program implementers to funders. We recommend that the final decision be made by the stakeholder ultimately using the evidence generated for decision-making. Ideally, the research team will work with the partners taking a cocreation approach, meaning that both the researchers and the partner will apply the framework and collectively design a learning approach to be measured. At a minimum, the research team must collaborate with the partners to ensure that all relevant information is collected to apply the criteria below. These criteria can also be used to assess the need for adaptation.

### Step 2: Select Activity and Calibrate the Level of Rigor

Figure 1 visualizes a generalized set of adaptive learning or RF activities, here, using the nomenclature that our team commonly uses to distinguish between activities. Some methods are generally more rigorous than others; for example, an impact evaluation is more rigorous than formative research or lean testing. But just as each of the criteria exists on a continuum connected to certainty, each of our adaptive learning activities exists on a continuum connected to rigor. Adaptive learning activities are inherently flexible and can be designed to be more rigorous or less rigorous. We use the “best-fit line” from step 1 to guide our choice of activities and to calibrate the level of rigor of those activities once chosen. In this way, we match the adaptive learning method to the learning question and not the other way around (i.e., we take our question and go in search of a method rather than start with a method in mind and go in search of a question).

In step 2, we use the “best-fit line” from step 1 to guide our choice of activities and to calibrate their level of rigor.

To use this framework effectively, it is important to recognize that certainty is dynamic over the course of an engagement, and its assessment should be revisited following each learning activity. Similar to the importance of returning repeatedly to a TOC as a living document, it is important to continue returning to this framework to adjust the dials in close collaboration with stakeholders. Moving the dials will change the best-fit line, suggesting which adaptive learning activities to take on next, as well as the associated level of rigor. The framework can be used to increase the sensitivity of researchers to what kind of measurement is needed, when it is needed, and for what purpose. The choice of adaptive learning approaches is a choice—and this framework provides considerations for both the measurement approach and the approach to adaptation.

### A Note on Feasibility

Feasibility considerations, such as budget, team capacity, timelines, stakeholder priorities, accountability to funders, and local context, also factor into decision-making when it comes to research methods. Once a method is chosen, these factors are often the driving force behind the final decision about how rigorous (or not) the implementation of a particular method will be. For example, a structured experiment may be the appropriately rigorous method for answering a learning question. But if the budget is limited, then researchers may end up implementing a cheaper, less rigorous experiment than they would ideally like to conduct given the research question. While the framework in this article is meant to explicitly introduce rigor considerations into decision-making in new ways, in some circumstances, researchers may need to give higher priority to these other feasibility considerations. In some cases, these factors may outweigh the final assessment done through the framework to determine the appropriate level of rigor.

We present 3 case studies that describe how our team applied this framework to get rigor right.

## 1. COVIDACTION RESILIENT HEALTH SYSTEMS: INFORMING COACHING SUPPORT WITH ADAPTIVE LEARNING

In 2020, the UK's Foreign, Commonwealth & Development Office funded dozens of small, short-term grants to test innovators' novel responses to the COVID-19 pandemic through an initiative called COVIDaction. Nine of these innovators whose work focused on building more resilient health systems were selected to receive coaching support from R4D. We supported these grantees in identifying opportunities to quickly validate their TOC and adapt implementation to achieve the goals set out in their grant applications.

### Adaptive Learning Methods

To assess the level of certainty for Step 1 of our framework, R4D worked with each grantee to form a long-term vision, near-term outcomes, a list of priorities, and key assumptions. Grantees had been selected for their local expertise and for the greater maturity of their innovations in the hopes that this would lead to a more rapid response to COVID-19. But the novel nature of COVID-19 dramatically changed the context everywhere, and even mature innovations were forced to adjust to the new circumstances. Partners were familiar with the environment but were learning to respond to COVID-19 (context) and introducing new components to long-tested programs (maturity). Grantees were trying to reach new constituents in many cases, so they just wanted a general understanding of their openness to new technology, such as telehealth apps, and would adapt the tools based on that learning (precision). Each grantee was operating with pace (urgency) to respond to the rising pandemic. The grantees had 6 months to design and implement their activities, meaning they would have to make decisions quickly using whatever evidence was available.

Our process of gaining insight into implementation challenges and testing assumptions was key to understanding each COVIDaction grantee's contribution to more resilient health systems.

**FIGURE 2:**
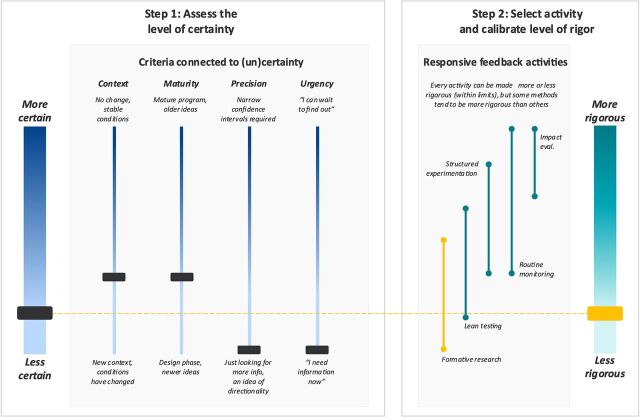
Applying the Framework to COVIDaction Resilient Health Systems Abbreviation: eval., evaluation.

Given how low we were on the certainty continuum, we decided that formative research was the most appropriate adaptive learning or RF activity, as shown in the yellow line in Figure 2. We wanted to generate as much evidence for grantees as we could in the shortest amount of time possible so that grantees would have relevant information to help them navigate the new COVID-19 context. As grantees sought to launch their innovations, we linked them to a diverse network of experts to provide technical assistance and facilitated a series of peer learning events. With each engagement, we documented practical and actionable lessons along each grantee's learning journey, collected formative evidence of what was working and what was not through light-touch monitoring activities, and built out related MEL activities. This process of gaining insight into implementation challenges and testing assumptions was key to understanding each grantee's contribution to more resilient health systems. In an environment of uncertainty, evidence of all kinds had the potential to inspire new insights, and formative research gave us the flexibility to facilitate learning in multiple ways.

### Reflections

#### This Framework Helped Us Make the Most of a Challenging Timeline and Capacity Constraints

Grantees found this framework actionable and relevant to their work. They learned about their own innovation, from each other, and from experts as a result of the responsive coaching that emerged from applying this framework. However, there is no question that dividing our coaching resources among 9 innovators over less than a year provided challenges not faced in the case studies that follow. For example, only 1 of our grantees was able to experiment with lean testing during the coaching period, but with more time, others would have likely benefited from such an opportunity.

#### Provide Space to Test, Fail, and Learn Quickly, While Recognizing That Scaling and Integration Is a Longer-Term Endeavor

While R4D provided critical financial support and technical assistance to the grantees at key points, we were only able to observe and support a brief portion of their overall scaling journey. As innovators scale, their future learning endeavors should follow a similar process of identifying levels of certainty and tailoring learning and rigor to those unique journeys.

#### Start With Country Needs, Priorities, and Demand

Many of our grantees started by recognizing a gap in their country's health system and innovating to fill it. This framework highlighted the need for country-led TOCs. To scale up these innovations and integrate them into the broader health system, it is critical to engage with a range of health system stakeholders early on to understand health system needs, priorities, and how technology and innovation can best support them. Government and health system actors can then identify and validate key health system gaps, diagnose their root causes, and evaluate and iterate on the innovations and technologies available to them.

### The Implementer Experience

The 9 participating implementers had useful feedback about the methodology, namely that it was best suited for early-stage innovators and that peer learning was the most valuable learning activity. Small teams needing deep coaching support were grateful for the hands-on approach. They noted that the experience “felt different” from the usual Foreign, Commonwealth & Development Office grant process. Implementers valued the peer learning, particularly those events which blended technical assistance from experts with opportunities to share and engage with peers on how to apply the expertise shared.

## 2. TUPAIA: SEQUENCING ADAPTIVE LEARNING WITH FORMATIVE RESEARCH AND LEAN TESTING

Our team engaged Tupaia, a data aggregation, analysis, and visualization platform that maps health systems in the Pacific Islands through a funding agreement with the Australian Department of Foreign Affairs and Trade.^20^ The Ministry of Health of a Pacific island nation was in the process of transitioning from paper-based to digital vaccine management and had hypothesized that Tupaia could support this transition.

We sequenced adaptive learning with formative research and lean testing to assist a Pacific island nation in transitioning from paper-based to digital vaccine management.

### Adaptive Learning Methods

Through a TOC validation exercise conducted with local stakeholders, we identified 3 areas of uncertainty: the accuracy of the new digital platform, the digital literacy of users, and the data needed for decision-making. Weaknesses in any of these areas would have undermined the program causal chain that starts with use of the Tupaia app and ends with improved health outcomes. We wanted to learn more about these areas so that any problems could be addressed and, as a result, the causal chain would be strengthened.

**FIGURE 3:**
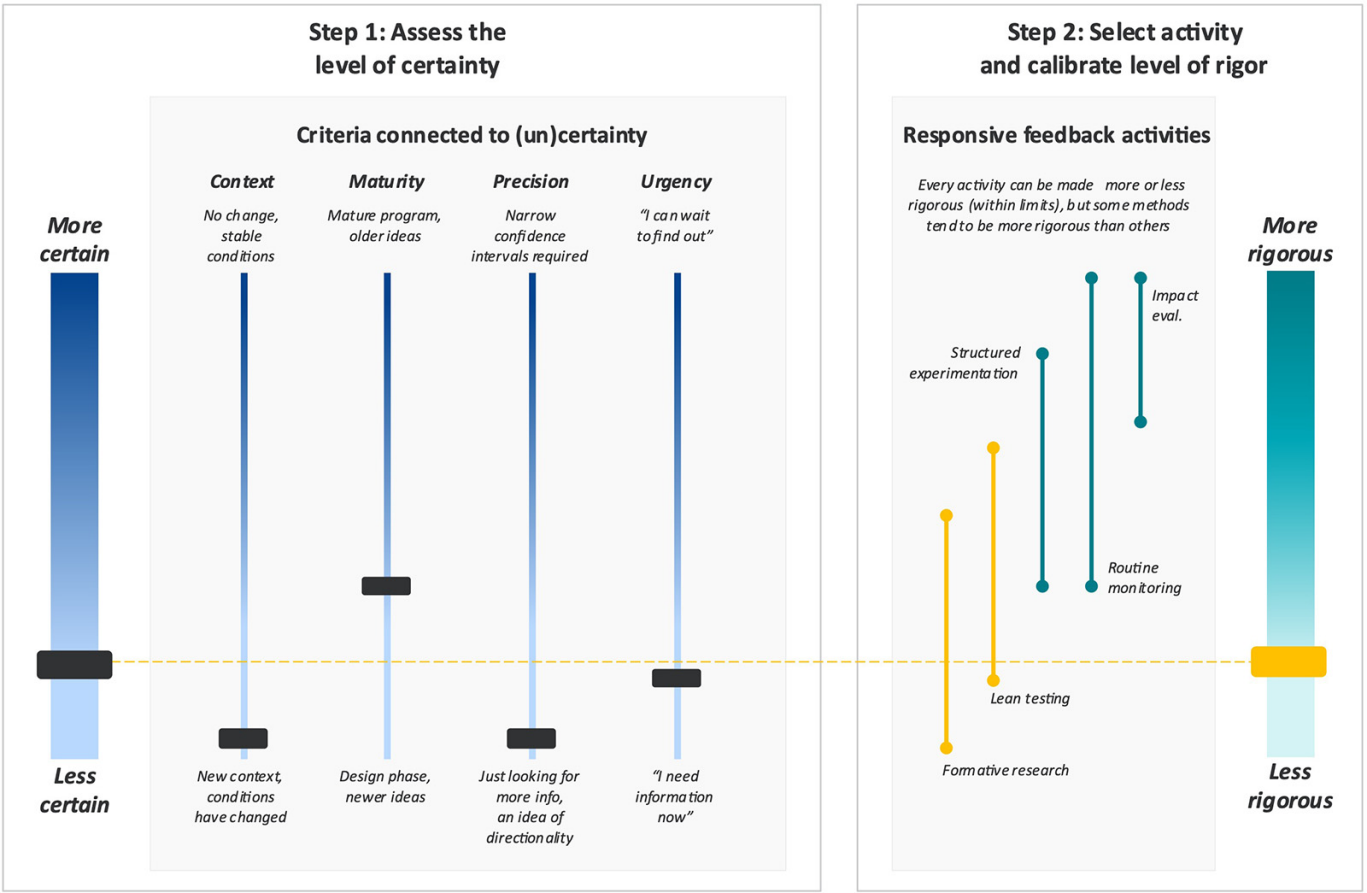
Applying the Framework to Tupaia Abbreviation: eval., evaluation.

Figure 3 shows our initial assessment of the need for rigor following the validation exercise. Tupaia was created by a social entrepreneur with deep experience in the Pacific. The technology had been tested (maturity), but the modules for vaccine tracking were brand new, and the app had not been used in the country where we were working (context). Early discussion with the entrepreneurs and health care workers revealed that several other challenges to effective vaccine management were potentially inhibiting Tupaia's effectiveness. This, combined with the fact that we had far more questions than answers, meant that any evidence we could collect was going to be valuable (precision). We also had limited time on our engagement (urgency), so we opted for low-rigor methods—formative research and lean testing—that would offer insights quickly about the context and challenges they would face (Figure 3).

For our formative research activities in country, we produced a systems map that visually laid out all the people and processes that were important to vaccine use and use of the Tupaia app. With the map, we were able to see all the ways that these parts of the systems intersected, which helped us identify areas of high and low certainty. R4D and Tupaia's implementation team workshopped these findings and developed a plan to iteratively test solutions to improve data quality, encourage data use by Ministry of Health and clinic staff, and support decision-making by Ministry of Health leaders. To improve data quality, we collected qualitative data through a series of key informant interviews and conducted spot checks, which showed the underlying data displayed on the platform was inaccurate. In response, we worked closely with the software designers and the health care team to ensure errors were spotted and corrected.

The formative research helped to fill in gaps in our knowledge about the context and gave us a general idea of how the app was functioning. We then conducted lean testing to focus on how the app is used by users. Through direct observation and a short quiz, we assessed users' digital literacy. This quickly revealed an important insight: users across the health care system had no trouble making sense of data visualizations but were less comfortable using digital technology itself. Therefore, we focused additional lean testing on providing in-person and telephonic technical support to 10 health care workers that helped us understand the level of investment needed to support health care workers in their use of the digital platform at scale.

Ensuring data quality and the ability of key stakeholders to engage with the technology were prerequisites for decision-makers to be able to use the data from the Tupaia platform when making decisions. The Tupaia team provided targeted support to decision-makers, and we conducted qualitative interviews with 10 key decision-makers before and after that support, which helped identify promising cases where data can be used for decision-making. We then recommended that those use cases be included in the health department's standard operating procedures to ensure long-term use. By taking this phased approach to adaptive learning in which we narrowed the areas of low certainty over time, we saw an overall improvement in the quality of the intervention.

By taking a phased approach to adaptive learning in which we narrowed the areas of low certainty over time, we saw an overall improvement in the quality of the intervention.

### Reflections

#### Lower-Rigor Approaches Are Most Relevant When Certainty Is Low

Across the 4 dimensions of the framework, the Tupaia intervention was close to the low end of the spectrum. An adaptive learning approach gave us the chance to run small-scale tests of potential interventions to increase the chance that scarce resources were used on approaches that would work. By testing varying levels of in-person technical assistance for health workers, we were able to determine the level of support needed for a nationwide scale-up plan.

#### Timing Matters

A brief implementation delay allowed us to share findings from formative research before the program was rolled out so that the immunization module of the digital platform could be built with the latest evidence. Getting data into the hands of decision-makers at the right time can be the difference between having an impactful partnership and merely having an interesting dataset.

#### Real-Time Data Aggregation, Plus Assumption Busting at the Outset of the Project, Is a Powerful Combination

Tupaia's premise—to aggregate and visualize data sources for better decision-making—is inherently appealing. But it also presumes a need for these services and presumes users will have the ability to understand and translate data visualizations into action. Assumptions undergird even our best ideas, and checking those assumptions through a right-fit evaluative process that reflects the certainty can save time and resources.

### The Implementer Experience

The Tupaia team provided feedback at the end of this engagement and identified 3 key lessons.

- **The combination of real-time data analysis and early testing was valuable.** The team felt that some of the thinking could have been done without a codified methodology but that the methodology made all of the assumption busting and data analysis greater than the sum of its parts.
- **Lean testing solutions is a time- and resource-efficient way to make decisions.** The team was new to the lean testing concept and saw some promising ways of working that they took forward beyond the engagement with the adaptive learning team.
- **At the pilot stage, adaptive learning is preferred to traditional M&E.** The team was initially skeptical of adaptive learning support based on past experience with third-party evaluators. However, the adaptive learning activities quickly revealed a solutions-oriented, trusted partnership that helped the team test and validate their TOC.

## 3. FAMILY CARE FIRST CAMBODIA: RIGHT-SIZING RIGOR FROM FORMATIVE RESEARCH TO STRUCTURED EXPERIMENTATION

Cambodia has experienced a rapid increase in the number of children placed in residential care institutions (RCIs) or private group living arrangements for children, even when most of the children have 1 or both parents living. This has significant effects on children's physical, social, emotional, and cognitive development. In response, the U.S. Agency for International Development (USAID) launched Family Care First (FCF) Cambodia in 2014 to address the rapid rise in number of these RCIs, the expansion of which was placing more children at risk for unnecessary separation. FCF consisted of over 30 Cambodia-based nongovernmental organizations contracted by USAID that were engaged in safely reducing the number of children living outside of family-based units in the country. There was strong agreement between the nongovernmental organizations and USAID on the intended impact of the FCF initiative, but numerous questions remained about how best to achieve that impact.

### Adaptive Learning Methods

Our guiding research and learning questions focused on approaches to social and behavior change communication among communities and international donors. We sought to determine the most promising activities to prevent unnecessary family-child separation in Cambodia and, of the proposed activities, the areas that could benefit from additional testing.

We sought to determine the most promising activities to prevent unnecessary family-child separation in Cambodia.

**FIGURE 4:**
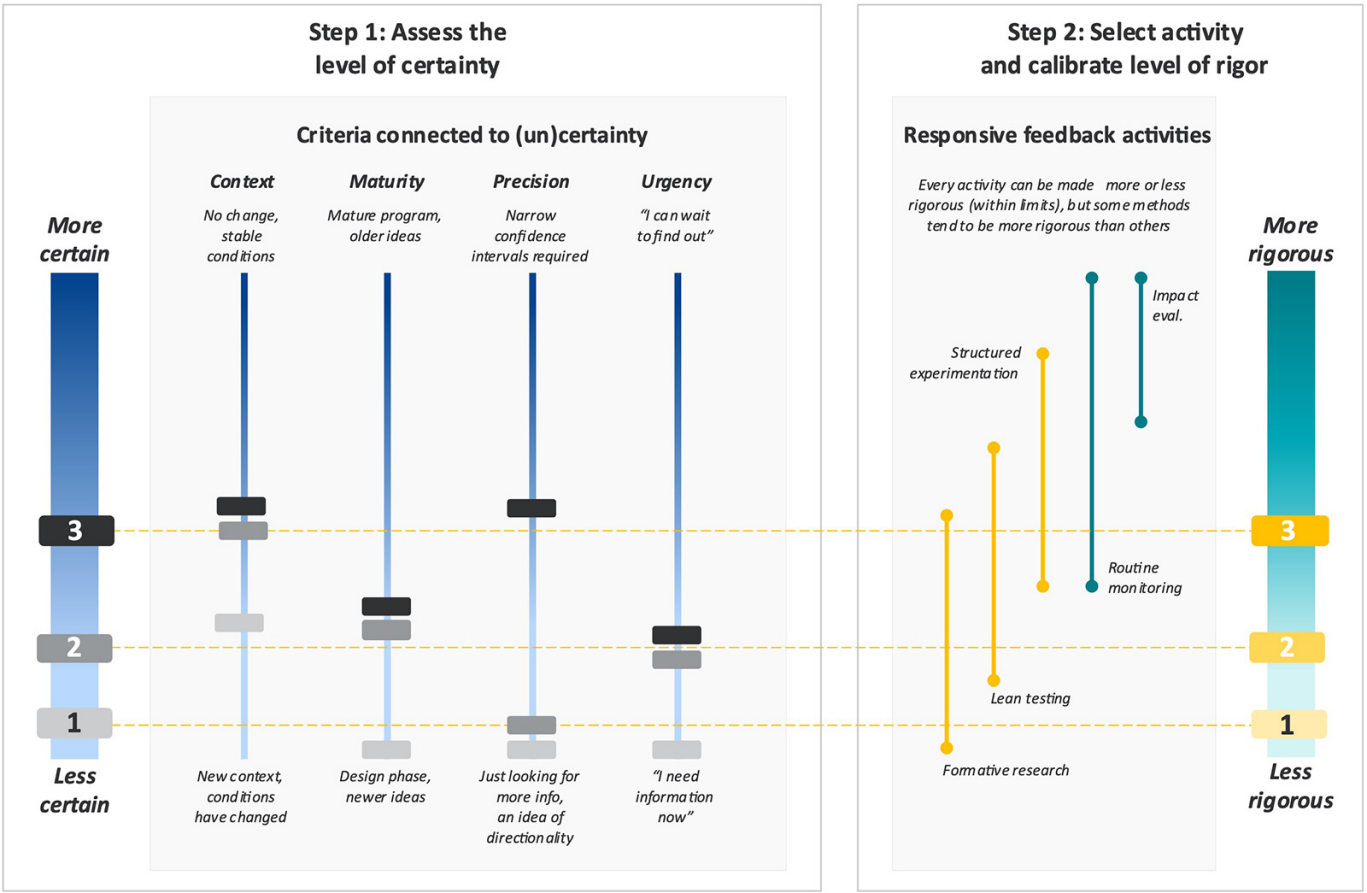
Applying the Framework to Family Care First Cambodia Abbreviation: eval., evaluation.

To do so, we conducted multiple sequential adaptive learning activities alongside design and implementation activities. This created a cyclical process of assessing certainty and conducting adaptive learning activities at one level of rigor, then taking the learning from those activities and reassessing certainty and conducting adaptive learning activities at another level of rigor. Through each successive adaptive learning feedback loop, the activities became more rigorous as our certainty in the design increased. Figure 4 demonstrates how rigor increased alongside increased certainty in the design.

#### Adaptive Learning Feedback Loop 1

In the first phase of work activities (see markers labeled “1” in Figure 4), the team implemented a TOC codesign and validation process with those that knew the program and the context best to identify priority. While our partners had an acute understanding of the development challenge, the proliferation of RCIs signaled that the context was evolving (context). This was the first time the partners had all worked together within a collective impact model framework, using a structured process to align 40+ implementing organizations to a common agenda, shared measurement, and mutually reinforcing activities; this required aligning on priorities across many diverse perspectives (maturity). The partners were eager to get started with implementation. With so many significant open questions about which interventions would work best, we knew that our first set of RF activities would not and could not settle on precise answers (precision). We needed to collect some basic evidence first, and we needed to do it quickly (urgency).

Through this process and by drawing on the existing evidence base, we were also able to focus on behavior change as a critical area of low certainty within the causal pathway for FCF Cambodia's TOC. With limited knowledge about how to engage stakeholders effectively, we conducted formative research to determine which social and behavior change communication campaign messages resonated with caregivers to change their attitudes and perceptions around RCIs. We tested posters and messages with a small sample and then held focus group discussions with respondents after viewing these messages for feedback. Additionally, this first feedback loop's formative research gave us our first inputs for designing a structured experiment.

#### Adaptive Learning Feedback Loop 2

As we gained more insight into the types of messages that might be effective and as the collective impact model of learning gained traction, our assessment of the level of certainty within maturity and context went up (see markers labeled “2” in Figure 4). This led us to consider more rigorous adaptive learning activities, like lean testing. For this activity, we drafted positive versus negative messages and used an A/B testing format to understand potential effects of the messages.

#### Adaptive Learning Feedback Loop 3

With 2 feedback loops complete, we now had more answers and fewer open questions (see markers labeled “3” in Figure 4). We had a much better understanding of the context (context). And having tested the social and behavior change communication messages directly, we had some idea of what might work. It was time to determine just how well the ideas might work. This raised our assessment of precision needed. As a result of these shifts in certainty, our next adaptive learning activity was to conduct a structured experiment. This meant completing dedicated baseline and endline surveys and introducing randomization, with certain communities randomly selected to receive certain messages through varied mediums. This allowed for more direct estimation of causal effects using advanced econometric methods.

Throughout the engagement, we conducted periodic workshops in which all stakeholders came together to discuss learnings and brainstorm the implications of the findings. These sessions were designed to combine research findings with the tacit knowledge of key stakeholders to continually reassess our level of certainty in the design and, therefore, determine the level of rigor for subsequent learning activities.

### Reflections

#### The FCF Cambodia Pilot Combined Lower-Rigor Activities With Higher-Rigor Activities Once Additional Certainty Was Achieved

The experience demonstrated what it means in practice to “crawl the design space,” using feedback loops to ensure that learning and adaptation inform ongoing design decisions.

#### Selecting Activities and Calibrating the Level of Rigor Requires Significant Engagement From Implementing Partners and the Adaptive Learning Team

Of the 3 case studies, FCF Cambodia was the most extensive, and we advanced through different stages of the framework throughout our engagement. Our team embedded a study coordinator with the implementing partners to ensure that we effectively captured their tacit knowledge and their assessment of certainty in the design. This also helped to ensure that our learning activities were aligned with implementer activities and that we were able to choose the appropriate level of rigor for each engagement. This required significant engagement from both the implementing partners and the adaptive learning team, given the importance of getting rigor right for each feedback loop.

#### Each Cycle of Feedback Offered Multiple Options for How to Move Forward

Cocreation was critical in selecting the path forward. The research team concluded each phase of engagement with many follow-on activity options for the implementers and USAID to consider. The selection process revolved around the components of our rigor framework, balancing stakeholder perspectives on what was most urgent and most feasible within existing constraints—using cocreation and participatory engagement to narrow in on the final option. The research team followed the lead of the implementers, prioritizing their research and learning questions to ensure that the research was utilization-focused and applicable to their pressing concerns.

### The Implementer Experience

Both implementing partners engaged in feedback activities noted how the evidence generated truly informed key programmatic design decisions. They pointed to the importance of having someone from the research team based in country to facilitate between implementation teams and technical research teams and questioned whether they would have achieved the same level of success without that. They also flagged that their deep involvement in designing the learning questions and carrying out data collection activities strengthened their capacity in adaptive learning methods, with one partner independently carrying out lean testing for other activities. However, they also noted that a participatory and cocreative process like this required significant time investments—much more than anticipated—and that this may not be feasible for all situations.

### Limitations

The framework outlined above requires deep contextual knowledge and strong partnerships to engage in cocreation. While some adaptive learning methods may be completed quickly, particularly when compared to more rigorous methods, none of the methods are necessarily inexpensive or “easy.” Learning is a complex task, and so in virtually any form, it is going to require large investments of time and expertise from all stakeholders involved.

Implementing any single method necessarily reveals tradeoffs with respect to scale, broader applicability, and budget. With limited funds, programs are often forced to choose between doing one—and only one—highly rigorous learning activity that precisely estimates causal impact and doing several less rigorous learning activities that yield insights on several elements of a program design. The rigor framework in this article is designed to help navigate these decisions. But it is notable that resource constraints may preclude subsequent rigorous evaluation when the framework initially points to lower-rigor adaptive learning activities.

The framework is also not foolproof. Deciding where to set the dials and knowing where to draw the “best-fit line” requires systematic thinking. But it also requires nuanced thinking—thinking that considers the strength of the evidence and the strengths—and perhaps, more importantly, the limitations—of the methods. Expert input is likely necessary to do both the systematic and nuanced thinking.

The framework is also not a guarantee of success in identifying a workable design. Indeed, we outline in more than one of the case studies discussed how our learning came only after multiple attempts. Our first conjectures about how to evaluate program success, test assumptions, or conduct experiments needed adjusting. As such, the framework should be considered as a guide to facilitate discussions around how to balance rigor and learning in individual adaptive learning feedback loops, to be continuously revisited to facilitate ongoing learning.

The framework should be considered as a guide and continuously revisited to facilitate ongoing learning.

## CONCLUSION

As applied to real-world evaluation problems, the framework has the potential to guide stakeholders through the process of assessing their programs to design relevant, timely, and iterative adaptive learning or RF activities. Focusing on the level of certainty is perhaps not an obvious place to start with evaluation, but it has resonated with our partners and produced actionable, relevant results. More research is needed on this framework moving forward.

The case studies within this article demonstrate the ways in which research and development practitioners can ensure that adaptive learning or RF activities strike the right balance between certainty about the program design and methodological rigor. Our framework offers an approach to determining the key considerations for getting rigor right, with the recognition that this requires a cocreation process involving a wide range of stakeholders. Just as there is no one-size-fits-all approach to M&E, the way in which decision-making happens using this framework also needs to be tailored to the individual situation. What is important is being clear on how and why certain decisions are being made in the tradeoffs between the various criteria. As the number of case studies increases, we suggest adapting and iterating on this framework to reflect the experiences of on-the-ground research and development practitioners in generating right-fit evidence. As the development community shifts away from a focus on quantitative rigor at the expense of other methods to a focus on learning and right-fit methods, there are opportunities to build in other dimensions like utility and feasibility to select the most appropriate methods.

## Notes

**Peer Reviewed**

**First published online:** March 22, 2023.
